# Improved *De Novo* Draft Genome Sequence of the Nocavionin-Producing Type Strain Nocardia terpenica IFM 0706 and Comparative Genomics with the Closely Related Strain Nocardia terpenica IFM 0406

**DOI:** 10.1128/MRA.00689-20

**Published:** 2020-08-20

**Authors:** Anina Buchmann, Harald Gross

**Affiliations:** a Department of Pharmaceutical Biology, Pharmaceutical Institute, University of Tübingen, Tübingen, Germany; b German Centre for Infection Research (DZIF), Partner Site Tübingen, Tübingen, Germany; Indiana University, Bloomington

## Abstract

We report an improved *de novo* draft genome sequence of the human-pathogenic strain Nocardia terpenica IFM 0706^T^. The resequencing unveiled that the genome size is larger than anticipated, reducing significantly the number of contigs and building a basis for comparison with the closely related strain *N. terpenica* IFM 0406.

## ANNOUNCEMENT

Strain IFM 0706^T^ (=JCM13033^T^=DSM44935^T^=NBRC100888^T^) was isolated in the 1990s from a nocardiosis patient and was originally identified as a Nocardia brasiliensis strain. Together with the strain N. brasiliensis IFM 0406 (1), it was recognized as a new species and reclassified as Nocardia terpenica, with IFM 0706 representing the corresponding type strain of this new species (2). Recently, IFM 0706^T^ was shown to produce the antibiotic nocavionin (3).

Within the course of our genome-driven investigations of *Nocardia* strains (4–6), we noted that a genome sequence of IFM 0706^T^ was available under its synonymous designation *N. terpenica* NBRC 100888^T^ (GenBank accession number BAGI00000000.1). However, an annotation was missing, and the genome is highly fragmented. In addition, in comparison with closely related strains (4, 7), we hypothesized that the genome size of 8.63 Mbp might be too small. In order to close the significant genomic gaps and to increase the genomic resolution focused on secondary metabolism, we resequenced the genome of strain IFM 0706^T^.

For genomic DNA isolation, a ZR Quick-DNA fungal/bacterial DNA miniprep kit (Zymo Research, Irvine, CA, USA) was used according to the manufacturer’s protocol, except that the vortexing step was reduced from 15 to 5 min and conducted at maximum speed. The DNA was sheared using a Covaris g-TUBE, and the genomic library was prepared according to the standard PacBio 6-kb multiplex protocol, followed by size selection with the BluePippin size selection system (Sage Science, Inc.). The library was sequenced on a PacBio Sequel instrument using v3.0 chemistry, including Sequel Polymerase v3.0 and one single-molecule real-time (SMRT) cell v3, resulting in 321,329 reads with a median read length of 4,523 bp. No quality filtering was conducted; however, subreads shorter than 50 bp were discarded. The remaining PacBio long reads were assembled using SMRTLink v7.0.1 and HGAP4 (8, 9). All software settings were kept at their default, except for the HGAP4 genome size estimate parameter, which was set to 9 Mbp. Overall, the reads were assembled to a 9,269,950-nucleotide draft genome at 142-fold coverage. The resulting sequence consists of 5 contigs with a G+C content of 68.52%. Gene functional annotation using PGAP v4.11 (10) identified 8,402 coding genes.

In summary, the resequencing of strain IFM 0706^T^ enabled us to increase the quantity (from 8.63 Mbp to 9.27 Mbp) and quality of genomic information, to significantly reduce the number of contigs (from 4,460 down to 5), to correct the G+C content (from 68.30 to 68.52%), and to provide the annotation.

**FIG 1:**
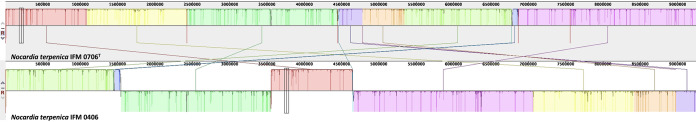
Mauve genome comparison between *N. terpenica* strains IFM 0706^T^ (top) and IFM 0406 (bottom). Each contiguously colored region is a locally colinear block (LCB), a region without rearrangement of homologous backbone sequence. LCBs identified by Mauve are color coded; links between LCBs are indicated by the thin colored lines. LCBs below a genome’s center line are in the reverse complement orientation relative to the reference DNA sequence. Unmatched regions within an LCB indicate the presence of a strain-specific sequence. The contigs are separated by red lines. The scale is in nucleotides.

Sequence alignment of IFM 0706^T^ with its closely related strain *N. terpenica* IFM 0406 (GenBank accession number LWGR00000000.1), employing Mauve snapshot_2015-02-25 (using the progressiveMauve algorithm) (11, 12), showed six large blocks of correspondence, confirming the overall relatedness of the two genomes, with the exception of multiple deletion/insertion segments (Fig. 1). In comparison to IFM 0406, the genome of IFM 0706^T^ contains an additional 95,215 bases (1.03%) and lacks 114,674 bases (1.24%). Further genomic indices were determined as follows: the average nucleotide identity (using autoMLST [13]), digital DNA-DNA hybridization, and difference in G+C content (Genome-to-Genome Distance Calculator v2.1, applying formula 2 [14]) between the strains were 100% (Mash distance, 0.0002), 99.50%, and 0.01% (68.51 versus 68.52%), respectively. This corroborated the similarities between the strains and confirmed that they belong to the same species. These findings are also reflected in the biosynthetic potential of both strains to produce secondary metabolites. Bioinformatics analysis using antiSMASH v5.1 (15) revealed that IFM 0706^T^ shares largely the same type of biosynthetic gene clusters (BGCs) with IFM 0406. In addition to 35 orphan BGCs, these include the BGCs for brasilinolide (16), terpenibactin (6), nocavionin (3), and brasilicardin A (1, 5).

### Data availability.

This whole-genome sequencing project has been deposited at DDBJ/ENA/GenBank under the accession number JABMCZ000000000. The corresponding raw sequencing data set has been registered in the NCBI SRA database under the accession number SRR11861893.

## References

[1] Shigemori H, Komaki H, Yazawa K, Mikami Y, Nemoto A, Tanaka Y, Sasaki T, In Y, Ishida T, Kobayashi J 1998 Brasilicardin A. A novel tricyclic metabolite with potent immunosuppressive activity from actinomycete *Nocardia brasiliensis*. J Org Chem 63:6900–6904. doi:10.1021/jo9807114. 11672311

[2] Hoshino Y, Watanabe K, Iida S, Suzuki S, Kudo T, Kogure T, Yazawa K, Ishikawa J, Kroppenstedt RM, Mikami Y 2007 *Nocardia terpenica* sp. nov., isolated from Japanese patients with nocardiosis. Int J Syst Evol Microbiol 57:1456–1460. doi:10.1099/ijs.0.64695-0. 17625175

[3] Wiebach V, Mainz A, Siegert M-AJ, Jungmann NA, Lesquame G, Tirat S, Dreux-Zigha A, Aszodi J, Le Beller D, Süssmuth RD 2018 The anti-staphylococcal lipolanthines are ribosomally synthesized lipopeptides. Nat Chem Biol 14:652–654. doi:10.1038/s41589-018-0068-6. 29915235

[4] Buchmann A, Eitel M, Koch P, Schwarz PN, Stegmann E, Wohlleben W, Wolanski M, Krawiec M, Zakrzewska-Cerwinska J, Mendez C, Botas A, Núnez LE, Morís F, Cortes J, Gross H 2016 High-quality draft genome sequence of the actinobacterium *Nocardia terpenica* IFM 0406, producer of the immunosuppressant brasilicardins, using Illumina and PacBio technologies. Genome Announc 4:e01391-16. doi:10.1128/genomeA.01391-16. 27979943 PMC5159576

[5] Schwarz PN, Buchmann A, Roller L, Kulik A, Gross H, Wohlleben W, Stegmann E 2018 The immunosuppressant brasilicardin: determination of the biosynthetic gene cluster in the heterologous host *Amycolatopsis japonicum*. Biotechnol J 13:1700527. doi:10.1002/biot.201700527. 29045029

[6] Chen J, Frediansyah A, Männle D, Straetener J, Brötz-Oesterhelt H, Ziemert N, Kaysser L, Gross H 2020 New nocobactin derivatives with antimuscarinic activity, terpenibactins A-C, revealed by genome mining of *Nocardia terpenica* IFM 0406. Chembiochem 21:2205–2213. doi:10.1002/cbic.202000062. 32196864 PMC7497119

[7] Herisse M, Ishida K, Porter JL, Howden B, Hertweck C, Stinear TP, Pidot SJ 2020 Identification and mobilization of a cryptic antibiotic biosynthesis gene locus from a human-pathogenic *Nocardia* isolate. ACS Chem Biol 15:1161–1168. doi:10.1021/acschembio.9b00763. 31697466

[8] Chin C-S, Alexander DH, Marks P, Klammer AA, Drake J, Heiner C, Clum A, Copeland A, Huddleston J, Eichler EE, Turner SW, Korlach J 2013 Nonhybrid, finished microbial genome assemblies from long-read SMRT sequencing data. Nat Methods 10:563–569. doi:10.1038/nmeth.2474. 23644548

[9] Chin C-S, Peluso P, Sedlazeck FJ, Nattestad M, Concepcion GT, Clum A, Dunn C, O'Malley R, Figueroa-Balderas R, Morales-Cruz A, Cramer GR, Delledonne M, Luo C, Ecker JR, Cantu D, Rank DR, Schatz MC 2016 Phased diploid genome assembly with single-molecule real-time sequencing. Nat Methods 13:1050–1054. doi:10.1038/nmeth.4035. 27749838 PMC5503144

[10] Tatusova T, DiCuccio M, Badretdin A, Chetvernin V, Nawrocki EP, Zaslavsky L, Lomsadze A, Pruitt KD, Borodovsky M, Ostell J 2016 NCBI Prokaryotic Genome Annotation Pipeline. Nucleic Acids Res 44:6614–6624. doi:10.1093/nar/gkw569. 27342282 PMC5001611

[11] Darling ACE, Mau B, Blattner FR, Perna NT 2004 Mauve: multiple alignment of conserved genomic sequence with rearrangements. Genome Res 14:1394–1403. doi:10.1101/gr.2289704. 15231754 PMC442156

[12] Darling AE, Mau B, Perna NT 2010 progressiveMauve: multiple genome alignment with gene gain, loss and rearrangement. PLoS One 5:e11147. doi:10.1371/journal.pone.0011147. 20593022 PMC2892488

[13] Alanjary M, Steinke K, Ziemert N 2019 autoMLST: an automated Web server for generating multi-locus species trees highlighting natural product potential. Nucleic Acids Res 47:W276–W282. doi:10.1093/nar/gkz282. 30997504 PMC6602446

[14] Meier-Kolthoff JP, Auch AF, Klenk H-P, Göker M 2013 Genome sequence-based species delimitation with confidence intervals and improved distance functions. BMC Bioinformatics 14:60. doi:10.1186/1471-2105-14-60. 23432962 PMC3665452

[15] Blin K, Shaw S, Steinke K, Villebro R, Ziemert N, Lee SY, Medema MH, Weber T 2019 antiSMASH 5.0: updates to the secondary metabolite genome mining pipeline. Nucleic Acids Res 47:W81–W87. doi:10.1093/nar/gkz310. 31032519 PMC6602434

[16] Chiu H-T, Weng C-P, Lin Y-C, Chen K-H 2016 Target-specific identification and characterization of the putative gene cluster for brasilinolide biosynthesis revealing the mechanistic insights and combinatorial synthetic utility of 2-deoxy-l-fucose biosynthetic enzymes. Org Biomol Chem 14:1988–2006. doi:10.1039/c5ob02292d. 26754528

